# 紫杉醇联合卡铂三周方案同步胸部放疗治疗不宜手术的局部晚期非小细胞肺癌疗效和安全性研究：一项来自单中心的回顾性研究

**DOI:** 10.3779/j.issn.1009-3419.2016.11.03

**Published:** 2016-11-20

**Authors:** 静 赵, 晓彤 张, 克 胡, 汉萍 王, 燕 徐, 晓燕 斯, 巍 钟, 霞 黄, 力 张, 孟昭 王

**Affiliations:** 100730 北京，中国医学科学院，北京协和医学院，北京协和医院呼吸科 Department of Respiratory Medicine, Peking Union Medical College Hospital, Chinese Academy of Medical Sciences and Peking Union Medical College, Beijing 100730, China

**Keywords:** 肺肿瘤, 局部晚期, 放化疗, Lung neoplasms, Locally advanced, Chemoradiotherapy

## Abstract

**背景与目的:**

对于局部晚期非小细胞肺癌（non-small cell lung cancer, NSCLC）同步放化疗是推荐的标准治疗。理想的化疗方案并未确立。本研究拟回顾性分析紫杉醇/卡铂（paclitaxel/carboplatin, PC）三周方案同步胸部放疗治疗局部晚期NSCLC的疗效和安全性，并与标准的依托泊苷/顺铂（cisplatin/etoposide, PE）方案进行比较。

**方法:**

回顾性分析北京协和医院2012年1月-2014年6月收治的局部晚期NSCLC患者共43例，其中15例接受PC三周方案同步胸部放疗，28例接受PE方案同步胸部放疗。比较两组患者的临床特征、疗效和不良反应。

**结果:**

全组患者：客观缓解率（objective response rate, ORR）为41.9%，疾病控制率（disease control rate, DCR）为90.7%，中位无疾病进展生存时间（progression-free survival, PFS）为10.6个月（95%CI: 7.4-13.8），中位总生存期（overall survival, OS）为19.2个月（95%CI: 15.3-23.1）。PC组和PE组在疗效上无统计学差异（ORR：33.3% *vs* 46.4%，DCR：86.7% *vs* 92.9%，*P*=0.638；PFS：6.6个月*vs* 12.2个月，*P*=0.389；OS：16.1个月*vs* 22.1个月，*P*=0.555）。不良反应可处理，两组均未发生治疗相关死亡。

**结论:**

PC三周方案同步胸部放疗治疗局部晚期NSCLC与标准PE方案疗效相似，不良反应可接受，在临床中可采用。

## Abstract (en)

**Background and objective:**

Standard care for patients with inoperable advanced non-small cell lung cancer (NSCLC) is concurrent chemoradiotherapy. The ideal concurrent chemotherapy regimen has not been determined. The aim of this study is to retrospectively analyze the efficacy and safety of concurrent radiotherapy with carboplatin/paclitaxel administrated every three weeks (PC three-week regimen) in inoperable advanced NSCLC and compare them with the results of cisplatin/etoposide.

**Methods:**

The 43 patients with inoperable advanced NSCLC receiving concurrent chemotherapy in Peking Union Medical College Hospital from January 2012 to June 2014 were enrolled and analyzed. Of them, 15 patients received carboplatin/paclitaxel with concurrent thoracic radiotherapy; the other 28 patients received cisplatin/etoposide. Clinical characteristic, efficacy and toxicity data were compared in these two groups.

**Results:**

For the overall population, the objective response rate (ORR) and disease control rate (DCR) were 41.9% and 90.7% respectively. The median progression free survival (PFS) was 10.6 months (95%CI: 7.4-13.8). And the median overall survival (OS) was 19.2 months (95%CI: 15.3-23.1). There were no significant differences in response rates (ORR: 33.3% *vs* 46.4%; DCR: 86.7% *vs* 92.9%, *P*=0.638), PFS (6.6 months *vs* 12.2 months, *P*=0.389), or OS (16.1 months *vs* 22.1 months, *P*=0.555) in either group. The adverse events were generally manageable and no treatment-related deaths occurred.

**Conclusion:**

Compared with PE, PC three-week regimen concurrent thoracic radiotherapy for inoperable advanced NSCLC has the similar efficacy and acceptable toxicity profile, which can be used in clinical setting.

非小细胞肺癌（non-small cell lung cancer, NSCLC）约占肺癌的85%，其中约1/3为局部晚期^[1]^。对于不可手术切除的局部晚期NSCLC，若患者体能状况良好，推荐的标准治疗为同步放化疗。然而，同步放化疗中放疗的最佳剂量和方式尚未确定，推荐的剂量波动于60 Gy-66 Gy之间。同步放化疗中最佳化疗方案亦未确定，但大多数方案均包含铂类药物。在Ⅲ期临床试验中使用过的方案包括：丝裂霉素、长春地辛联合顺铂^[2]^，依托泊苷联合顺铂（PE）^[3]^，长春花碱联合顺铂^[3]^，紫杉醇联合卡铂（PC）^[4]^、长春瑞滨联合顺铂^[5]^以及培美曲塞联合顺铂^[6]^。目前临床上使用最广泛的方案是PE方案（VP-16 50 mg/m^2^, d1-d5, d29-d33; DDP 50 mg/m^2^, d1, d8, d29, d36）和PC周疗方案（Paclitaxel 45 mg/m^2^，d1、d8、d15、d22、d29；Carboplatin曲线下面积=2，d1、d8、d15、d22、d29）。然而，PC周疗方案必然会导致患者更多次住院以及更大量预处理激素的使用，给临床治疗带来了一定的困难，因此，将PC周疗方案修改为PC三周方案（Paclitaxel 175 mg/m^2^，d1；Carboplatin曲线下面积=5，d1，每3周一次）联合胸部放疗，值得研究。本研究拟回顾性分析北京协和医院收治的，使用PC三周方案同步放疗治疗不适合手术的局部晚期NSCLC患者的疗效和安全性，并与传统的PE方案进行对比。

## 1 资料和方法

### 1.1 纳入和排除标准

纳入标准：①2012年1月-2014年6月北京协和医院肺癌中心收治的局部晚期NSCLC患者；②所有患者均接受PE或PC同步胸部放疗；③所有患者均经组织病理学或细胞学确诊为NSCLC，并按美国癌症联合委员会（American Joint Committee on Cancer, AJCC）第7版分期标准判定为IIIa期（不适合手术）或IIIb期。排除标准：①临床资料不完整者；②痰液细胞学诊断的NSCLC者；③具有影响生存的基础疾病，如活动性肺部感染、严重肺间质纤维化、不稳定心绞痛等。

### 1.2 化疗方案

PE组化疗方案为：依托泊苷VP-16 50 mg/m^2^，d1-d5，顺铂50 mg/m^2^，d1，d8，每28天1周期，同步放化疗结束后，巩固化疗2周期；PC组化疗方案为：紫杉醇175 mg/m^2^，d1，卡铂按照曲线下面积=5计算，d1，每21天1周期，同步放化疗结束后，巩固化疗2个-4个周期。

### 1.3 放疗方案

采用6MV X射线直线加速器，照射范围包括原发灶、同侧肺门及纵隔。放疗中位剂量为60 Gy，1.8 Gy/次-2.2 Gy/次，5次/周。所有患者均采用三维适形放疗或调强放疗。危及器官限量：脊髓最大剂量 < 45 Gy；肺V20≤28%；心脏V30 < 40%，V40 < 30%；食管V50 < 50%。所有患者放疗均是在第一个化疗周期内开始。

### 1.4 临床资料的收集

通过查阅病历，获取患者的临床基线特征及治疗信息。本研究收集以下数据：性别、年龄、吸烟史、病理类型、肿瘤-淋巴结-转移（tumor-node-metastasis, TNM）分期、美国东部肿瘤协作组（Eastern Cooperative Oncology Group, ECOG）评分、疗效、不良反应。

### 1.5 疗效评价

依照实体瘤疗效评价标准（Response Evaluation in Solid Tumors, RECIST）1.1版，分为完全缓解（complete response, CR）、部分缓解（partial response, PR）、疾病稳定（stable disease, SD）和疾病进展（progressive disease, PD）。CR和PR必须在4周后经影像学检查确认。无疾病进展生存时间（progression-free survival, PFS）定义为开始同步放化疗至首次记录的PD时间或死亡时间。总生存期（overall survival, OS）定义为开始同步放化疗至患者死亡的时间。随访截止时间至2016年5月30日。

### 1.6 安全性评价

按照不良事件常用术语标准（Common Terminology Criteria for Adverse Events, CTCAE）4.0版评价不良反应。

### 1.7 统计学方法

采用SPSS 21.0统计学软件进行数据统计学处理。计量资料采用中位数或均数描述，计数资料采用率表示。率的比较采用卡方检验或秩和检验。生存数据采用*Kaplan-Meier*分析并进行*Log-rank*检验。所以统计检验均为双侧检验，*P* < 0.05表示差异有统计学意义。

## 2 结果

### 2.1 患者基线特征

本研究共纳入患者43例，其中接受PC方案进行同步放化疗15例，接受PE方案进行同步放化疗28例。全组患者男性36例（83.7%），女性7例（16.3%）；中位年龄59.5岁（41岁-75岁）；有吸烟史者28例（65.1%），既往有吸烟史4例（9.3%），从不吸烟者11例（25.6%）；ECOG 0分-1分者38例（88.4%），ECOG 2分者5例（11.6%）；鳞癌占大多数，共31例（72.1%），腺癌10例（23.3%）以及大细胞癌2例（4.7%）；分期上IIIa期8例（18.6%），IIIb期35例（81.4%）。PC组和PE组在性别、年龄、吸烟状态、ECOG评分、病理类型和分期均无统计学差异（表 1）。

### 2.2 治疗情况

PC组：总体完成计划治疗率60.0%（9/15）。15例（100%）患者均完成了与放疗同时进行的前2周期化疗；12例患者（80%）完成了放疗，中位放疗剂量为60 Gy（56 Gy-62 Gy），3例患者（20%）未完成放疗，中位放疗剂量为22.5 Gy（20 Gy-25 Gy）；对于放疗方式，4例（26.7%）患者采用三维适形放疗，11例患者（73.3%）采用了调强放疗；5例患者（33.3%）未行巩固化疗，10例患者（66.7%）完成了后续巩固化疗，中位化疗周期数3个（1个-4个）。

PE组：总体完成计划治疗率50.0%（14/28）。28例（100%）患者均完成了与放疗同时进行的前2周期化疗；22例患者（78.6%）完成了放疗，中位放疗剂量为60 Gy（58 Gy-62 Gy），6例患者（21.4%）未完成放疗，中位放疗剂量为35 Gy（34 Gy-40 Gy）；对于放疗方式，12例（42.9%）患者采用三维适形放疗，16例患者（57.1%）采用了调强放疗，均进行了组织不均匀性校正，处方剂量为95%计划靶区接受的剂量；11例患者（39.3%）未行巩固化疗，17例患者（60.7%）完成了后续2程的巩固化疗。

### 2.3 疗效评估

全组：18例（41.9%）PR，21例（44.8%）SD，4例（9.3%）PD，ORR为41.9%，DCR为90.7%；PC组：5例（33.3%）PR，8例（53.3%）SD，2例（13.3%）PD，ORR为33.3%，DCR为86.7%；PE组：13例（46.4%）PR，13例（46.4%）SD，2例（7.1%）PD，ORR为46.4%，DCR为92.9%。PE组和PC组在疗效上无统计学差异（*P*=0.638）。

### 2.4 复发转移情况

全组：18例（41.9%）出现局部进展，7例（16.3%）出现远处转移，10例（23.3%）同时出现了局部进展和远处转移，仍有8例（18.6%）在随访中未出现进展。PC组：5例（33.3%）出现局部进展，4例（26.7%）出现远处转移，4例（26.7%）同时出现了局部进展和远处转移，仍有2例（13.3%）在随访中未出现进展。PE组：13例（46.4%）出现局部进展，3例（10.7%）出现远处转移，6例（21.4%）同时出现了局部进展和远处转移，仍有6例（21.4%）在随访中未出现进展。PC组和PE组，在复发进展模式上无统计学差异（*P*=0.496）。

### 2.5 PFS

至末次随访时，全组患者：35例患者已进展，中位PFS 10.6个月（95%CI: 7.4-13.8）；PC组患者：12例患者已进展，中位PFS 6.6个月（95%CI: 0-14.3）；PE组患者：23例患者已进展，中位PFS 12.2个月（95%CI: 9.7-14.7）。两组采用*Log-rank*检验，无统计学差异（*P*=0.389）。两组患者的PFS曲线如图 1所示。

**1:**
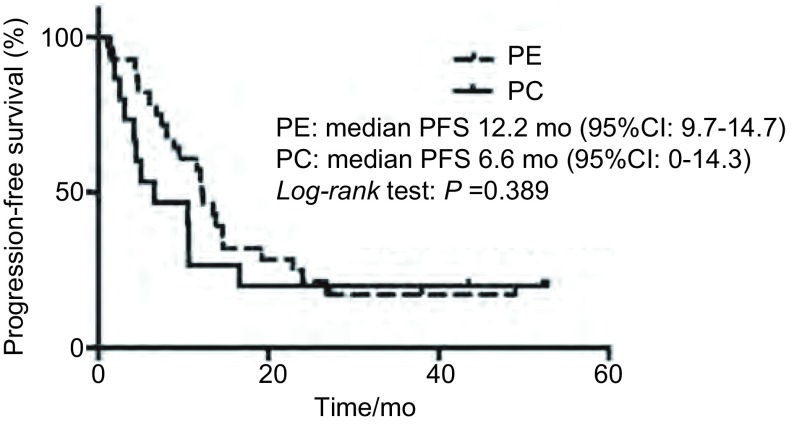
患者的无进展生存曲线 *Kaplan*-*Meier* estimates of progression-free survival (PFS) for the different groups

### 2.6 OS

至末次随访时，全组患者：34例患者已死亡，中位OS 19.2个月（95%CI: 15.3-23.1）；PC组患者：11例患者死亡，中位OS 16.1个月（95%CI: 12.1-20.1）；PE组患者：23例患者已死亡，中位OS 22.1个月（95%CI: 17.1-27.1）。两组采用*Log-rank*检验，无统计学差异（*P*=0.555）（图 2）。

**2:**
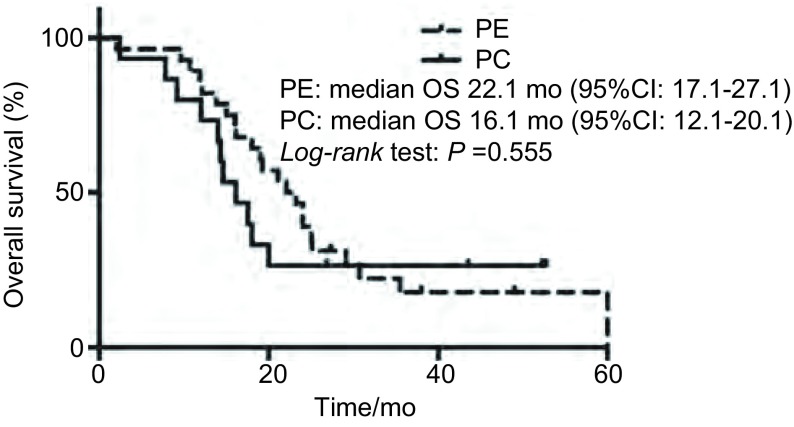
患者的总生存曲线 *Kaplan*-*Meier* estimates of overall survival (OS) for the different groups

### 2.7 不良反应

全组患者：总不良反应发生率为40/43（93%），其中3级-4级严重不良反应发生率为11/43（25.6%）；PC组：总不良反应发生率为15/15（100%），其中3级-4级严重不良反应发生率为4/15（26.7%），常见的不良反应包括：骨髓抑制6/15（40%），手麻3/15（20%），乏力3/15（20%）；PE组：总不良反应发生率为23/28（82.1%），其中3级-4级严重不良反应发生率为7/28（25%），常见的不良反应包括：骨髓抑制8/28（28.6%），放射性食管炎6/28（21.4%），恶心/呕吐3/28（10.7%）。两组不良反应发生情况详见表 2。尽管PC组骨髓抑制副作用发生率高于PE组（40% *vs* 28.6%），但无明显统计学差异（*P*=0.507）。

## 3 讨论

对于不可手术的局部晚期NSCLC，单纯放疗患者5年生存率仅为3%-10%。1996年CALGB8433研究报道^[7]^对于局部晚期NSCLC序贯化放疗优于单纯放疗，5年生存率为17%和6%（*P*=0.012）。随后RTOG9410^[3]^以及2010年发表的*meta*分析^[8]^显示，对于局部晚期NSCLC，同步放化疗较序贯放化疗能够进一步提高患者生存率和局部控制率。基于此，各国NSCLC治疗指南将同步放化疗推荐为不可手术的局部晚期NSCLC的标准治疗。

**1:** 43例患者临床资料 Clinical characteristics of the 43 study patients

Characteristics	All (*n*=43), [*n* (%)]	PC (*n*=15), [*n* (%)]	PE (*n*=28), [*n* (%)]	*P*
Gender				0.215
Male	36 (83.7)	11 (73.3)	25 (89.3)	
Female	7 (16.3)	4 (26.7)	3 (10.7)	
Age [median (range)]	59 (41-75)	58 (41-75)	59.5 (43-70)	0.842
Smoking status				0.176
Current	28 (65.1)	7 (46.7)	21 (75.0)	
Former	4 (9.3)	2 (13.3)	2 (7.1)	
Never	11 (25.6)	6 (40.0)	5 (17.9)	
ECOG				0.660
0	10 (23.3)	3 (20.0)	7 (25.0)	
1	28 (65.1)	11 (73.3)	17 (60.7)	
2	5 (11.6)	1 (6.7)	4 (14.3)	
Histology				0.129
Squamous	31 (72.1)	8 (53.3)	23 (82.1)	
Adenocarcinoma	10 (23.3)	6 (40.0)	4 (14.3)	
Large cell	2 (4.7)	1 (6.7)	1 (3.6)	
Stage				0.419
Ⅲa	8 (18.6)	4 (26.7)	4 (14.3)	
Ⅲb	35 (81.4)	11 (73.3)	24 (85.7)	
Response				0.638
PR	18 (41.9)	5 (33.3)	13 (46.4)	
SD	21 (44.8)	8 (53.3)	13 (46.4)	
PD	4 (9.3)	2 (13.3)	2 (7.1)	
Progression mode				0.496
Local progression	18 (41.9)	5 (33.3)	13 (46.4)	
Metastatic progression	7 (16.3)	4 (26.7)	3 (10.7)	
Both	10 (23.3)	4 (26.7)	6 (21.4)	
Non-progression	8 (18.6)	2 (13.3)	6 (21.4)	
ECOG: Eastern Cooperative Oncology Group; PR: partial response; SD: stable disease; PD: progressive disease; PC: paclitaxel/carboplatin; PE: cisplatin/etoposide.				

**2:** 血液学和非血液学毒性反应

Adverse events	PC (*n*=15), [*n* (%)]	PE (*n*=28), [*n* (%)]
Neutropenia/thrombocytopenia	6/15 (40.0)	8/28 (28.6)
3-4	4/15 (26.7)	5/28 (17.9)
1-2	2/15 (13.3)	3/28 (10.7)
Esophagitis		
1-2	2/15 (13.3)	6/28 (21.4)
Nausea/vomiting		
1-2	2/15 (13.3)	3/28 (10.7)
Fatigue		
1-2	3/15 (20.0)	None
Neuropathy		
1-2	3/15 (20.0)	None
Skin rash		
1-2	1/15 (6.7)	1/28 (3.6)
Hemoptysis		
1-2	None	1/28 (3.6)
Elevated alanine aminotransferase		
3-4	None	2/28 (7.1)
Radiation pneumonitis		
1-2	None	2/28 (7.1)

本单中心回顾性研究显示：与PE方案相比，采用PC三周方案同步胸部放疗治疗局部晚期不可手术的NSCLC，两种治疗方式疗效相似，中位OS分别为16.1个月和22.1个月，无明显差异。这一结果与既往研究结果一致，使用PC周疗同步胸部放疗，OS波动于16个月-22个月^[9-14]^；使用PE方案同步胸部放疗，OS波动于15个月-36.5个月^[2, 9-11, 15-18]^。其他化疗方案，如吉西他滨/顺铂^[19]^、培美曲塞/顺铂^[6]^或最新方案，如靶向药物（厄洛替尼^[20]^、吉非替尼^[21-23]^、西妥昔单抗^[24-26]^）联合放化疗、抗血管生成药物（贝伐珠单抗^[27]^、内皮抑素^[28]^）、蛋白酶体抑制剂^[29]^联合放化疗等，治疗组OS波动于11个月-25.2个月，与常用方案（PE或PC）相比，亦无明显差别。因此，对于局部晚期NSCLC，同步放化疗的疗效似乎达到了一个平台，联合更新药物，如免疫治疗或寡转移病灶更加积极的局部治疗，或许能进一步提高局部晚期NSCLC的疗效，此需进一步研究。

本研究显示：PC三周方案组或PE方案组，两组实际完成计划治疗率相近。导致放疗中断的原因主要是病灶出现空洞，担心大咯血而停止，其他因素还包括放射性肺炎和食道炎。导致化疗中断的原因主要是严重骨髓抑制以及患者依从性。尽管PC三周方案同步放疗骨髓抑制副作用明显高于PE方案，但统计学无差异。至于放射性食管炎、放射性肺炎以及恶心/呕吐等不良反应发生率两者相似。同时少数患者出现了与使用紫衫类药物相关的神经毒性，但不严重，均为1级-2级，未出现因不良反应所致的停药。两组不良反应的发生情况与既往研究^[9-11]^报道较为一致。但国外研究^[30, 31]^显示，紫杉类药物能够明显增加放射性肺炎的发生，这点与本研究结果不甚一致，可能与PC组患者更高比例采用了调强放疗技术或PC组患者太少，选择性偏倚明显有关。

本研究的不足之处在于为回顾性研究，入组例数少，而且鳞癌比例偏高，PC组巩固化疗高于PE组，这些可能导致偏倚。因此，需要进一步积累样本量以及进行设计良好的随机研究，以得到更加可靠的结果。

综上，本回顾性研究结果显示，对于局部晚期不可手术的Ⅲ期NSCLC，采用PE或PC三周方案同步放疗，两者疗效相似。紫杉醇和卡铂三周方案联合放疗亦是一种安全的治疗手段，可临床使用或进一步研究。
